# A model of contact-induced language change: Testing the role of second language speakers in the evolution of Mozambican Portuguese

**DOI:** 10.1371/journal.pone.0212303

**Published:** 2019-04-25

**Authors:** Anna Jon-And, Elliot Aguilar

**Affiliations:** 1 Centre for the Study of Cultural Evolution, Stockholm University, Stockholm, Sweden; 2 School of Humanities and Media Studies, Dalarna University, Falun, Sweden; 3 Dept. of Biology, University of Pennsylvania, Philadelphia, PA, United States of America; Newcastle University Institute for Health and Society, UNITED KINGDOM

## Abstract

Language change is accelerated by language contact, especially by contact that occurs when a group of speakers shifts from one language to another. This has commonly been explained by linguistic innovation occurring during second language acquisition. This hypothesis is based on historical reconstructions of instances of contact and has not been formally tested on empirical data. In this paper, we construct an agent-based model to formalize the hypothesis that second language speakers are responsible for accelerated language change during language shift. We compare model predictions to a unique combination of diachronic linguistic and demographic data from Maputu, Mozambique. The model correctly predicts an increased proportional use of the novel linguistic variants during the period we study. We find that a modified version of the model is a better fit to one of our two datasets and discuss plausible reasons for this. As a general conclusion concerning typological differences between contact-induced and non-contact-induced language change, we suggest that multiple introductions of a new linguistic variant by different individuals may be the mechanism by which language contact accelerates language change.

## Introduction

Language contact is the encounter between speakers of different languages. While all languages change over time, language contact is widely believed to accelerate the pace of language change [1]. This is especially the case when contact results in a group of people adopting a new language, a process known as language shift. History is rife with examples of language shift, such as the spread of vulgar Latin across the Roman Empire in Western Europe, or the adoption of Arabic during the Muslim conquests in the Middle East and North Africa. Given these and myriad other examples, language shift is likely to have affected numerous languages across the world and throughout history. A widely accepted explanation for the increased change is that language shift involves large proportions of new speakers who acquire the growing language as a second language (L2). These speakers are presumed to introduce novel linguistic forms at a greater rate than do native (L1) speakers as a result of the second language acquisition (SLA) process. The novel forms are then transmitted to later generations of both L1 and L2 speakers causing the language to change [1–4]. If true, this idea predicts that the pace of linguistic change should be correlated with the rate of introduction of L2 speakers in the population.

Large numbers of L2 speakers have been associated with linguistic simplicity, such as the lack of redundancy or grammatical regularity [5, 6]. Recent studies based on synchronic linguistic data have shown global relationships between proportions of L2 speakers, or population size as a proxy for this, and morphosyntactic complexity as well as lexical diversity [7–10]. Adult learner’s simplifications have also been observed in experiments involving artificial language learning [11]. While these results support the hypothesis that second language acquisition is involved in contact-related change, the lack of diachronic quantitative data has made it difficult to study the mechanism by which SLA accelerates change on a population level. In this sense, the hypothesis has not been formally tested.

In this paper, we use simulations to model language change during language shift, and compare the results to rare diachronic linguistic and demographic data from Maputo, Mozambique, where language shift from several Bantu languages to Portuguese has been going on since the country achieved its independence from Portugal in 1975. During the colonial period, use of the Portuguese language was restricted to Portuguese colonizers and Afro-Portuguese urban dwellers, and was spoken by few other Mozambicans [12]. In the years following independence, expansion of the educational system and the migration of Bantu-speaking rural Mozambicans to cities in large numbers resulted in a dramatic increase in the usage of Portuguese. The number of speakers of Portuguese is still increasing throughout the country, and especially in the capital Maputo. As a consequence of language shift and language contact, several linguistic features in Maputo Portuguese have undergone changes during this period [13–16]. Diachronic quantitative data on linguistic variation during a period of intense language contact and change is very rare. From Maputo, we have two comparable sets of recordings from 1993-4 and 2007, and as we have good reasons to believe that the innovations we are studying have diffused after the country’s independence in 1975, we may estimate a point where the innovations were not present. Due to regular censuses including information on languages, we can also estimate the proportions of first and second language speakers during this period. This unique combination of data provides us with with the necessary minimal conditions to test our model.

Simulation models are a useful way to explore the mechanisms by which new linguistic forms spread in a population through innovation and interaction, overcoming the difficulty of scarce data and empirical constraints. Mathematical and computational models have been employed in the field of language evolution to explain a number of phenomena, for example the emergence of grammatical or phonological systems in human language [17–19]. Simulation methods can be combined with empirical data to test the explanatory power of models. For example, Baxter et al. introduced a model for contact-related change whose predictions were compared to demographic and linguistic data from the evolution of New Zealand English [20]. More recently, Jansson et al. presented a model for the formation of creoles, another contact-related phenomenon, and applied their results to the case of Mauritian Creole [21]. Using agent-based simulations and demographic data from the colonization of Mauritius, Jansson et al. were able to accurately model how speakers of multiple languages converged on the current structure of Mauritian creole.

We modify the creole formation model of Jansson et al. to allow for both the introduction and propagation of novel variants in a population of L1 and L2 speakers of the same language. In order to test the hypothesis that variation is introduced during the second language acquisition process, we allow only L2 speakers to introduce the novel variant while entering the population. We then simulate this model in a fixed and an expanding population. Next, we run the model with demographic parameters based on the growth of the Portuguese speaking population of Maputo, Mozambique in the years 1975-2007, a period of ongoing language shift from Bantu languages to Portuguese, and compare the model results with data on changes in two grammatical features of Mozambican Portuguese. We test two models, one simpler model where individuals may introduce the novel variant only at the very moment of entering the population, and another where the second language acquisition process lasts for seven years allowing individuals to introduce novel variants on multiple occasions. The motivation for testing the two variants is that the representation of demographic dynamics and changing proportions of second language learners is central to the model. There is reason to suspect that the representation of second language learning as instantaneous with parameters set to real demographic data may be misrepresentative, and that representing second language learning more realistically as a process that has effects during a number of years, may generate different predictions. Our results show that the second model is in better agreement with the data.

## Materials and methods

### Model of language change

Our aim was to model the evolution of a linguistic feature due to repeated interactions in a group of L1 and L2 speakers, where only the latter group could introduce novel variants into the language. For example, if the linguistic feature is subject-verb agreement, as in “I/you/we/they go; he/she/it goes” we can imagine that some L2 speakers, while acquiring the language, reduce this paradigm to the simpler “I/you/we/they/he/she/it go” (Note: this is similar to what happens in Mozambican Portuguese, and one of our data sets is on reduced subject-verb agreement, *see* below). In this case, the loss of the verbal suffix, and use of an already existing simpler form, represents the novel variant introduced by L2 speakers, while the maintenance of the suffix represents the conservative variant. Linguistic innovation does not necessarily imply inventing completely novel variants, but rather commonly consists in a new form-meaning mapping where both form and meaning already exist in the language but have not previously been associated with each other. Thus, introducing a novel variant in the model may imply adding a linguistic element, but it may also imply removing or replacing one, the latter two being more common forms of linguistic change.

The frequencies of usage of the conservative and novel variants change over time due to repeated interactions between speakers. To model the interactions between speakers, we adapted the model of linguistic interaction introduced in Jansson et al. [21] for creole formation. This model allows individuals to interact and update their knowledge according to the outcomes of interactions. For a given language feature, an individual, *i*, is characterized by a discrete usage distribution, *p*_*i*_ ≔ {*p*_*i*1_, *p*_*i*2_, …, *p*_*in*_}, where *p*_*ik*_ is the probability of using variant *k* in an interaction, and $\sum_{k=1}^{n}p_{ik}=1$. At discrete time steps, a round of interaction occurs: each individual encounters another and both choose to utter a variant according to their respective usage distributions. In the encounter between agents *i* and *j*, let the variant uttered by *i* be *u*_*i*_, and the variant by *j* be *u*_*j*_. After an interaction, an individual *i* updates her probability of using each variant in the following way,

$$\begin{array}{cc} p_{ik}^{\prime }= & p_{ik}+\left(1-p_{ik}\right)l,\;\text{if}\;u_{j}=k \end{array}$$ (1)

$$\begin{array}{cc} p_{ik}^{\prime }= & p_{ik}-p_{ik}l,\;\text{if}\;u_{j}\neq k\; \end{array}$$ (2)

These equations simply state that an agent *i* will increase her probability of using a variant if her partner in the interaction uses it, and that increase will come at the expense of all other variants equally. The parameter, 0 ≤ *l* ≤ 1 is the learning weight and determines the importance an agent gives to each interaction; thus, high *l* means that agents change their probabilities of usage substantially after just one interaction, while low *l* means that they update only slightly. In the absence of innovation the model results in convergence on a stable form of the language.

### Demographic change and growth in the simulations

We allowed demographic change to occur through births of L1 speakers, recruitment of L2 speakers (*see* Table A in S2 Text), and deaths of both types. Each newborn L1 speaker selected two agents at random as ‘parents’ and averaged their usage distributions to obtain its own. This represents the idea that native speakers will enter the population with a limited number of linguistic model (e.g. their two parents). Each recruited L2 speaker averaged the entire population to obtain its initial usage distribution, representing the idea that a second language learner will interact with many individuals when first acquiring the language. However, with probability *μ*, instead of averaging the population, a newly recruited L2 speaker assigned the novel variant a probability of 1 and all other variants a probability of zero. This allowed L2 speakers to introduce novel variants on entering the population. We set *k* = 1 to be the existing variant, and initialized the simulation with *p*_*i*1_ = 1 for all agents to represent the idea that initially only the standard variant is present.

We allowed agents to complete *T* rounds of interactions before demographic change occurred. In the fixed population model, *dN* individuals are selected for death and replaced with either a “birth” (producing a L1 speaker), or “recruit” (producing a L2 speaker), according to the rates *b* and *r*, respectively. In the case of the expanding population simulations, deaths, births, and recruitments all occurred with rates *d*,*b*, and *r*, respectively. The overall growth rate was given by *b* + *r* − *d* = .05 for all expanding population runs.

We briefly note that the model described above is neutral with respect to interactions, in the sense that interactions do not favor either variant. To demonstrate this, let ${\bar{p}}_{k}=\frac{1}{N}\sum_{i=1}^{N}p_{ik}$, the population mean use of variant *k*. The expected change in the average frequency of usage of the variant is then $E[{\bar{p}}_{k}^{\prime }-{\bar{p}}_{k}]=0$, (*see* S1 Text). However, due to unidirectional mutation, extinction is guaranteed for the existing variant, presuming that recruitment rate is always greater than zero, since ${\bar{p}}_{1}=0$ is an absorbing state, while the novel variant can always be rescued from extinction by recurrent mutation. In the rest of the paper we will be concerned with the *rate* at which the novel variant increases in mean frequency of usage.

We ran simulations for 100 years, with 365 interactions per year, with demographic change occurring at the end of each year. For the comparison with language change data from Maputo, Mozambique, we ran simulations based on demographic data from the years 1975-2007 (*see* S2 Text).

#### Maputo linguistic and demographic data

We chose to compare the model predictions to changes in one morphological feature (third person plural verbal agreement) and one syntactic feature (choice of preposition for indicating destination). Morpho-syntax is generally more resistant to change than lexicon or phonology [22], which implies that change in these features during a restricted period of time is less likely to occur without the influence of language contact. Furthermore, reduced verbal morphology and prepositional change have previously been appointed as typical results of language contact in African and Brazilian varieties of Portuguese [14, 23–27].

For both phenomena, one novel linguistic variant has diffused at the expense of one conservative variant. The two novel linguistic variants are already present in other contexts in Portuguese, the innovations thus consisting in the use of the forms in novel contexts. In the case of verbal agreement, the conservative variant consists in a verb expressing agreement with the third person plural subject by a suffix, as in example (1), where the verb *fazer* (*to* *do*) in present tense has the plural suffix *em*. The novel variant is the lack of the plural suffix on the verb, the verb assuming a morphologically simpler form, as in example (2), where the verb *dever* (*to* *owe*/*must*) has no plural suffix in present tense. The morphologically simpler form coincides with the verbal form used with a third person singular subject. For the prepositional change, the conservative variant consists in the use of the preposition *a* (commonly *to* in English) to indicate destination, following a verb of movement. This is exemplified in (3), where the present tense form *vou* of the movement verb *ir* (*to* *go*) is followed by the preposition *a*, here contracted with the definite article *o*. The novel variant consists in the use of the preposition *em* (commonly *in* or *at* in English) as in example (4), where the same verb *ir*, here in infinitive, is followed by *em*, forming the word *no* contracted with the definite article *o*. The novel use of the preposition *em* in contexts of movement may be considered a simplification, as this innovation would reduce the number of locative prepositions if it went to completion. The examples are produced by speakers of Maputo Portuguese.

(1) *o que eles fazem para educação*
  ’what they do for education’
(2) *os homens não deve transar cabelo*
  ’the men should not braid their hair’
(3) *vou ao mercado*
  ’I go to the market’
(4) *eu costumo ir no hospital*
  ’I ususally go to the hospital’

There are good reasons to believe that these two novel variants have essentially diffused in Mozambique among Mozambican speakers after the country’s independence in 1975. The novel variants have rarely or never been observed in European mainland Portuguese. There are accounts of the use of the shorter verbal form in third person plural contexts in Portugal, but they are generally reduced to a few examples found in large corpora [28]. In the few quantitative accounts to be found on variable verbal inflection in historical or contemporary European Portuguese, consisting in analysis of pre-classical Portuguese texts [28] and more recent data from spoken metropolitan Lisbon Portuguese [29], the frequency of the reduced verbal form is consistently between 0 and 1%. As for the preposition *em* in European Portuguese, there is general consensus that it occurs in contexts of space but not of direction or movement, where the preposition *a* is used, historically as well as presently [30–32].

It seems thus reasonable to presume that the novel variants we are studying were not present or were present at a negligible level in the Portuguese that was introduced in Maputo by Portuguese settlers from the 15th century and onward. Portuguese was spoken almost exclusively by the settlers in Mozambique until the country’s independence in 1975, and was diffused within the original local population mainly after independence when it was adopted as the country’s only official language and there was a massive expansion of education [33]. We have no reason to believe that the Portuguese spoken by settlers in Maputo before independence differed radically from that spoken in Portugal, since new settlers kept arriving in increasingly large numbers, especially during the last 25 years of colonization [34, 35]. At the time of independence, when Portuguese was still predominantly spoken by the colonizers, the conservative variants would thus have been used almost exclusively. Even though novel variants may have been present at low levels, it is very unlikely that any substantial diffusion of these variants occurred before the massive introduction of Portuguese as a second language among Mozambicans after independence. For the above reasons we assume in our model that in 1975 only the conservative variant is present.

We have two comparable data sets of semi-spontaneous oral Maputo Portuguese from 1993 and 2007, where the frequency of the conservative and the novel variant is counted. The data sets were recorded within the frameworks of studies by Stroud and Gonçalves [23] and Jon-And [15]. For both linguistic features, the use of the novel variant increases during the period. For third person plural verbal agreement, the novel variant starts at a presumed zero in 1975, spreads to a sample mean of 10.6 percent in 1993 and reaches 20 percent in 2007. For destination prepositions, the conservative variant *a* is presumed to be used exclusively in 1975, while the novel variant *em* reaches a sample mean of 16.9 percent of the observed destination contexts in 1993, increasing to 25.7 percent in 2007. For details on data sets, analysis and relevant excerpts, *see* S3 Text and S1 Dataset.

The demographic data in Table 1 contains information on the number of L1 and L2 speakers in Maputo from censuses in the years 1980, 1997 and 2007. We can estimate these numbers for the year of independence, 1975, based on population data from the World Bank, and L1/L2 speaker estimates for 1952, 1955 and 1970 made by Firmino [12, 33].

**Table 1:** Demographic data on Portuguese speakers in Maputo, Mozambique.

year	L1 speakers	L2 speakers
1975	100*	20,000
1980	6,525	326,521
1997	241,709	599,438
2007	470,690	612,992

*Due to the massive emigration of Luso-Mozambicans around the time of independence, the number of L1 speakers in 1975 was estimated based on overall population growth and the recruitment rate.

For a rigorous test of the model, more than three chronological data points for each linguistic feature would be necessary. It is hard to find data that allows for quantifying linguistic variation at several comparable time points during ongoing language contact. Access to two chronological datasets recorded under similar conditions while being able to estimate the starting point for the novel variants is far from common, and combined with the demographic data on proportions of L1 and L2 speakers, this makes Maputo one of the best empirical examples for testing a model of language shift and change. While earlier studies have provided interesting information on the correlation between proportions of second language speakers and different measures of linguistic complexity, the kind of combined chronological data we are using is necessary to say something about the mechanism of change. Due to the scarcity of data we are unable to draw any strong conclusions from our model. However, considering that this is the first time a mechanism of contact-induced change is modeled and tested, we are interested not in an exact fit between model and data, but testing whether there is a qualitative fit, i.e. a correct prediction of an increase or a decrease of the variants between the given time points.

## Results

We were first interested in how the model parameters *r* (recruitment rate of L2 speakers), *μ* (innovation rate), and *l* (learning parameter) affected the spread of the novel variant in the population. For both the fixed and growing population models we ran the ten simulations at each constellation of parameter values for 100 model years (36500 rounds of interaction). We found that the rate of increase of the novel variant was most sensitive to *μ* and *r*, whose product determined the rate of introduction of the novel variant. The learning parameter *l*, did not significantly affect the outcome of simulations beyond leading to increased variability across sets of identically parameterized runs. In the following results we use the lowest learning rate of.05 for the following two reasons: 1) we expect the actual weight given to any one encounter to be small and 2) the lower learning rate means lower variance in the runs and sets a higher threshold for fitting the model to data.

**Fig 1:**
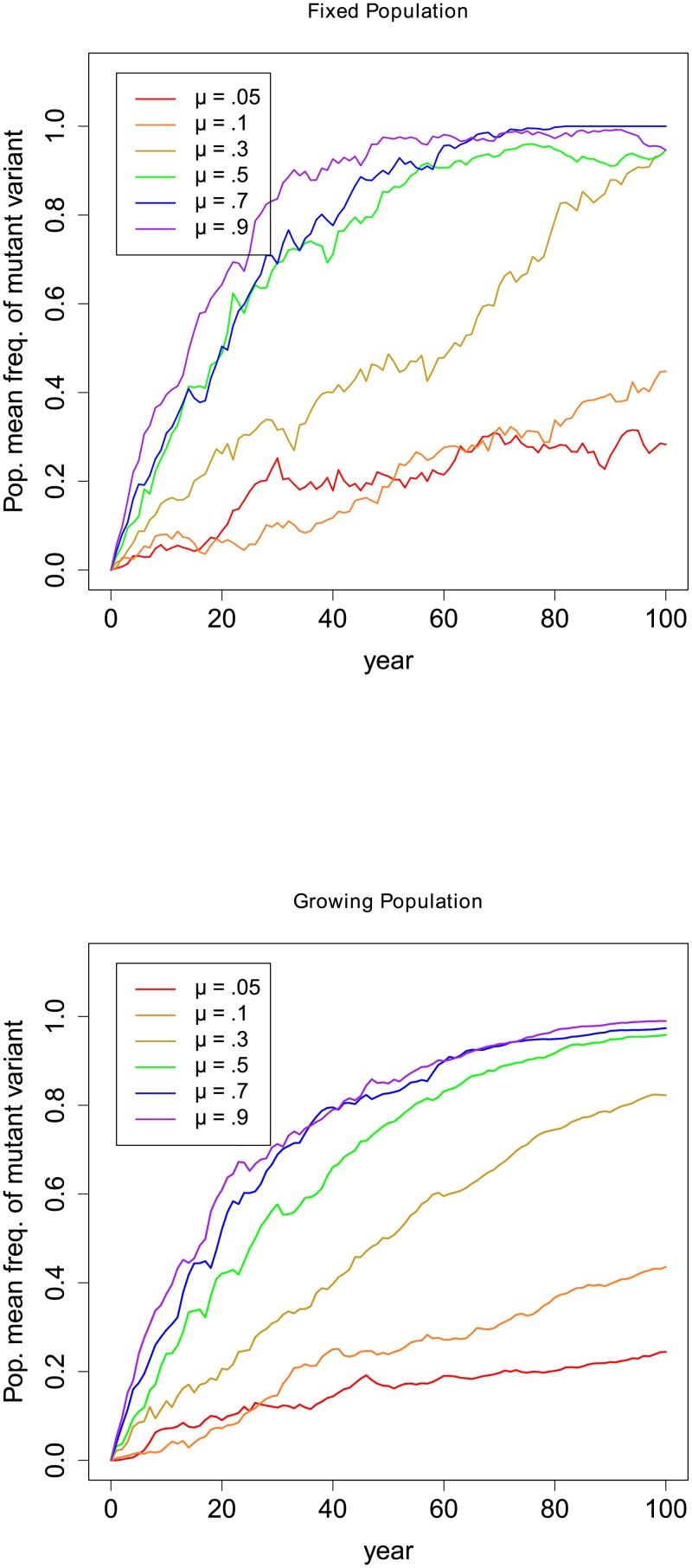
Change in the population mean usage of the mutant variant (averaged across ten runs) over 100 years. The other model parameter values are birth rate, b = .007, recruitment rate, r = .063, learning parameter, l = .1. For fixed population runs, N = 100, while for expanding populations, *N*_0_ = 100, with a growth rate of g = b+r-d = .05; *μ* is the mutation rate among L2 speakers. The trajectories of the expanding population means show more stability due to lower variance across runs.

Fig 1 shows the means of ten runs for each constellation of parameters. A notable difference between the fixed and expanding population runs is that the latter showed more stable mean trajectories. This results from the fact that for every model year after year 1 the expanding population models have a larger population, effectively increasing the sampling size of each run and reducing the variance across runs. This sampling effect is similar to that of genetic drift; for small populations the change in the mean use of the novel variant is likely to be more erratic.

Next we compared our runs parameterized by the Maputo demographic data to our usage data on Maputo Portuguese. We computed the 95% confidence intervals for each ensemble of runs (assuming the value of the mean frequency of usage for each run was drawn from a normal distribution, which was a good qualitative fit). We then computed the maximum likelihood estimate of the mean frequency of usage (i.e. probability of usage) of a novel variant for both datasets. In order to produce these maximum likelihood estimates from the data, we first assumed that each individual speaker’s use of the novel variant was binomially distributed; we then assumed that the parameter of each individual’s distribution was drawn from a beta distribution. The mean of this beta distribution represented the population mean frequency of usage of the novel variant. We checked to see whether a given parameter setting could a be a potential *fit* to one or both of the data sets by seeing if its 95% confidence intervals included the maximum likelihood estimate of the observed mean frequency of usage for the 1993 and 2007 data for those same years.

**Fig 2:**
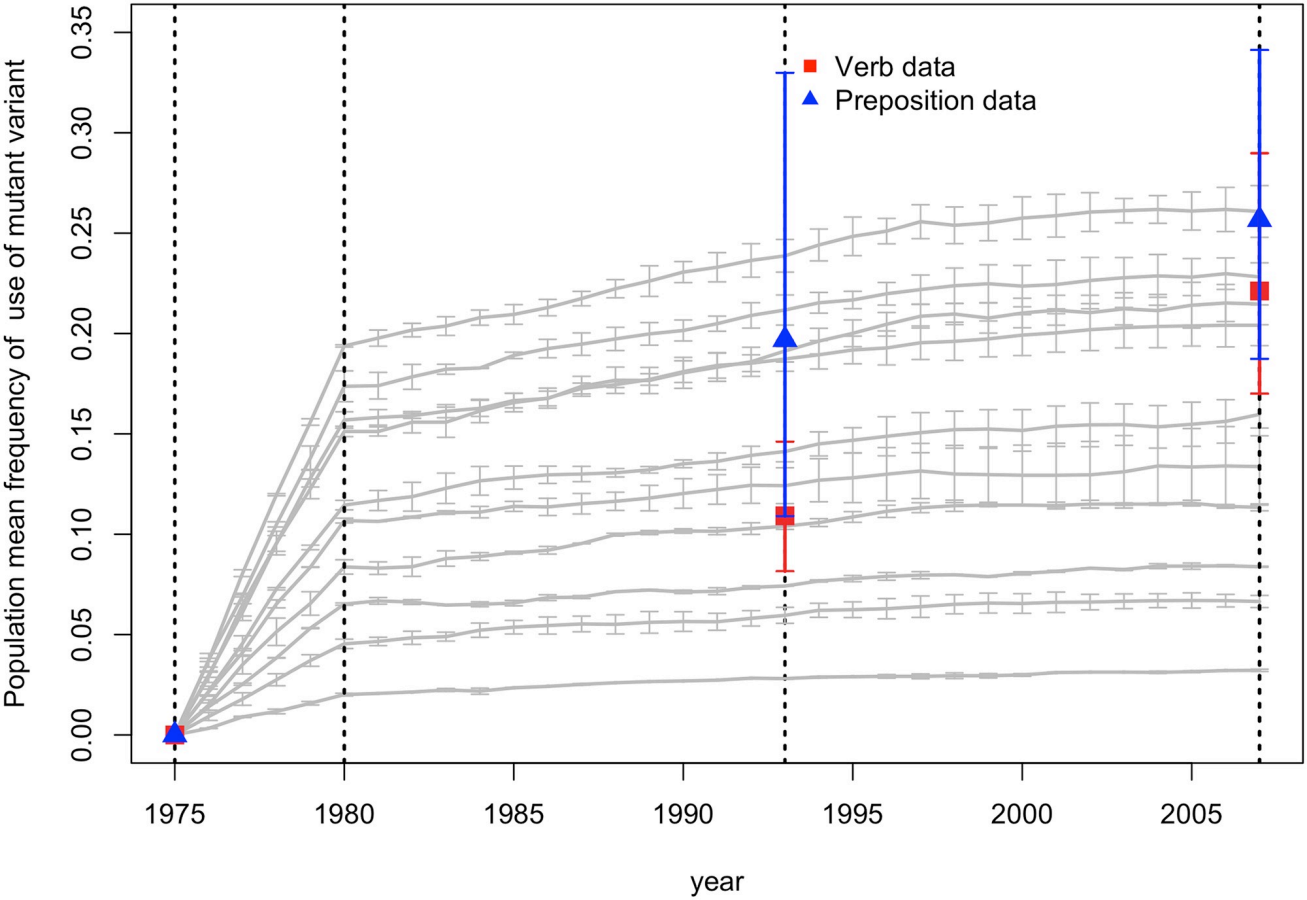
Comparison of verb and preposition maximum likelihood estimates of mean usage with the mean model trajectories (*l* = .05, *μ* = .01 −.1, with step sizes of.01). Both data sets show greater spread than predicted by the model in the third growth phase (1993-2007), though the slope of the preposition data is reduced in qualitative agreement with the model. The verb data actually shows an increase in the spread of the novel variant, which conflicts with the model predictions.

Fig 2 shows that none of the model runs met the criterion above. Generally, the model predicted a reduction in the rate of spread of the novel variant in each time period, as the rate of recruitment declined as well. Taking this into account, we made a modification to the initial model. Instead of only allowing incoming L2 speakers to introduce novel variants, we allowed all L2 speakers to spontaneously mutate at the start of each year for the first seven years after they first entered the population. This modification more closely resembles the reality of the SLA process, which occurs over a number of years. Furthermore, empirical findings on the effect of length of residence in SLA indicate that seven years is a good estimate for the duration of SLA [36, 37]. Except for this modification, the model remained unchanged. We refer to this model as Model 2.

**Fig 3:**
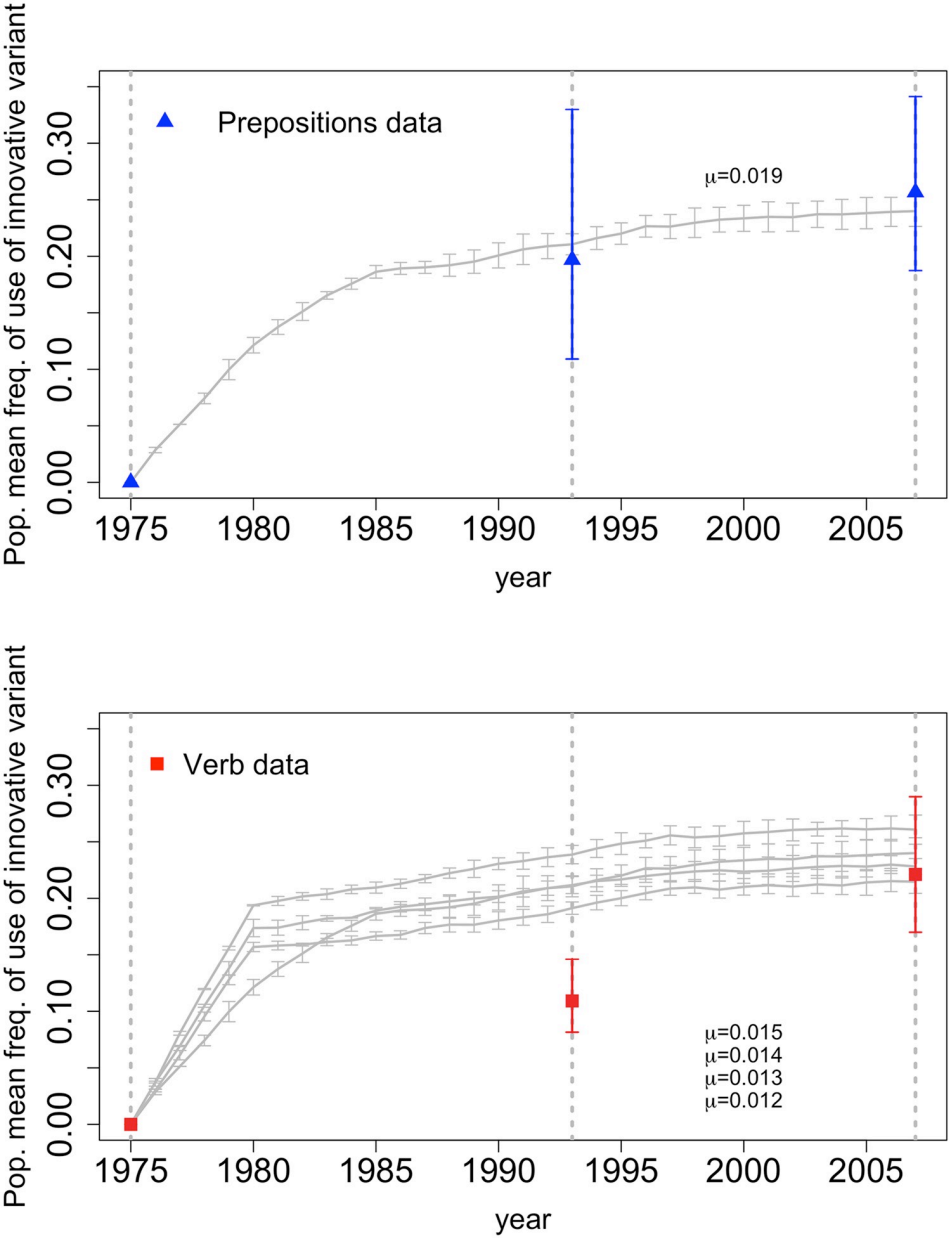
Comparison of verb and preposition usage data with the model 2 (SLA) mean trajectories. (Top) Multiple runs had confidence intervals that overlapped with the preposition use data; here we show the parameter setting with the minimum mean square error (*μ* = .019). (Bottom) No parameter values produced confidence intervals that overlapped with the verb data for both years. Here we include the run with the minimum mean square error (*μ* = .015).

The modified model also failed by the criterion of including the maximum likelihood estimates of the data within its 95% confidence intervals across a range of parameter settings for both data sets, *see* Fig 3. For the verb data there was no overlap between any of the model settings 95% confidence intervals and either of the 95% confidence intervals for the MLE estimates, as was the case for the previous model. However, we did observe overlap between the MLE estimate 95% confidence intervals for the preposition data and model confidence intervals for both the first and second model. We then selected which parameter settings in both models had the minimum mean squared distance from the maximum likelihood estimates of the preposition data; we ran 100 replicate runs each at these parameter settings. We fitted normal distributions to the simulated data from these replicates and used them to calculate the probability of obtaining a value from the simulation model equal to or more extreme than the maximum likelihood estimates from the data. In fitting these distributions, we assumed that the distributions of frequencies of usage were independent at each time step, conditioned on the underlying model. That is to say that, assuming the underlying model, we assumed that the normal distribution fitted to the simulated data at year 1993, was independent of the distribution fitted to the simulation at year 2007.

The results in Table 2 show that the model runs from the second model are a modest improvement on the probability of getting a value at least as extreme as the MLE estimate for the preposition data.

**Table 2:** Probabilities of both maximum likelihood estimates for the mean use of the novel preposition variants given the models.

	*P*(Prep data MLE 1, Prep data MLE 2|*Model*)
Model 1	0.056
Model 2 (SLA)	0.075

## Discussion

This paper represents the first time that a hypothesis about the mechanism that causes accelerated language change in language shift contexts has been formalized and tested. We modeled the introduction of novel variants due to an influx of L2 speakers in both a fixed and an expanding population. We then compared the model behavior with empirical data collected in Maputo, Mozambique in 1993 and 2007. The simpler model, where L2 speakers could only introduce a new variant on entering the population, showed a strong decline in the rate of spread of the novel variant after 1980, due to lower population growth after this year. This model correctly predicted an increased proportion of use of the two novel variants between the first and the second data point, as well as between the second and the third data point. Furthermore, it predicted a decline in the rate of spread between the second and third data point, which is a qualitative fit to the preposition usage. However, within the given confidence intervals, this model could not account for the changes observed in the data on verb forms or preposition usage. When we extended the period of second language acquisition to seven years in the model, and let the L2 speakers introduce the new variant on multiple occasions during this period, we saw modest improvement in model fit. Altogether, this is a promising result given that we only constrained the model by the demographic change of Maputo Portuguese speakers. However, the low number of data points and the relatively small size of our data sets makes it difficult to draw any strong conclusions concerning the validity of the model fit.

It must be reiterated that our model assumed no selective pressure on the linguistic form, only recurrent mutation. Given this assumption, the departure of the verb data from model predictions may be indicative of directed or selectional forces acting on this feature. For instance, the new verb forms may be easier to learn and/or produce (the conservative form is longer and the distinction between singular and plural form for third person implies a more complicated verbal conjugational paradigm) and so spread due to a bias among speakers to be more economical with their articulatory/cognitive effort. Learnability of a language has been shown to increase particularly in growing speech populations with many L2 speakers [38]. On the other hand, there is no reason to believe that the innovative preposition is more or less difficult to learn or produce than the conservative one and the prepositional change may thus evolve without any system-internal selective pressure essentially favoring one variant.

One limitation of our model is that it assumes that the learning parameters are the same for all individuals. This may not be true in the sense that L1 and L2 speakers may perceive one another’s different speaker status and respond differently in mixed interactions than in interactions among their own class of speakers. Different speakers may also have different biases towards conservative and novel variants, depending on factors such as their age or their L1/L2 status. Future work should explore the importance of varying learning parameters for interactions between the different speaker classes, as well as heterogeneous biases in a contact situation. Additionally, there may be heterogeneity in learning parameters across all individuals, regardless of speaker class.

Overall our model and simulations did demonstrate how with minimal assumptions, novel variants can be introduced and spread in a population, leading to the eventual extinction of the existing variant. Theoretical models of innovation and propagation of linguistic forms in a stable population have shown that specific conditions, in terms of biases towards novel variants in the population and/or network position of the innovator, are required for the novel variant to be successful [20, 39, 40]. These models represent the spread of a single innovation (introduced at one occasion by one speaker), thus accounting for language change in a context where no pressure from language contact is involved. This implies a low probability of spread for any innovation occurring in the population, representing a good general match for non-contact induced change. By contrast, our model assumes a homogeneously mixing population. This fact alone should not favor the spread of the novel variant. However, a more important difference between our model and previous work is the possibility of repeated introductions. This provides a sustained mutation pressure that, in the absence of directional forces, results in the eventual spread of the novel variant.

The assumption that several L2 speakers introduce the same new variant independently of one another represents the hypothesis that second language acquisition triggers certain general effects, such as for example morphological reductions. Experimental results show that L2 learners do simplify an artificial language, but when their output is used as the input of new generations of speakers, it does no result in the next generation’s language becoming less complex than the original language, but rather the opposite [11]. This may be due to learner’s use of different strategies of simplification, creating a highly variable input for next generation, in contrast with our model where there is only one novel variant that may be introduced. Our model is in agreement with our data where one novel variant replaces a conservative one, possibly because the novel forms already exist and are likely to be chosen in the need for simplification, combined with learner’s interaction and settlement for a common new variant at an early stage. Other recent experiments indicate that learners regularize and accommodate within the group when given the chance to interact [41]. This underlines the conclusion that selective pressures from learning seem to be a central characteristic for contact-induced change.

## Conclusion

We built a multi-agent simulation model to test the hypothesis that second language speakers introduce linguistic change in language shift. Our model was a modestly better fit when SLA-like processes were introduced.

At a general level, our model demonstrates how multiple introductions of a novel variant allow a linguistic innovation to spread in a population without any of the specific conditions required in models of non-contact-induced change being fulfilled. We thus suggest that multiple versus single introduction of novel variants may be a basic typological difference between contact-induced and non-contact-induced language change, able to explain how SLA increases language change in shift situations.

## Supporting information

S1 Text Mathematical details. (PDF)

S2 Text Demographic data. (PDF)

S3 Text Linguistic data. (PDF)

S1 Dataset Excerpts from datasets. (XLSX)
