# Supplementary material for: A model of contact-induced language change: Testing the role of second language speakers in the evolution of Mozambican Portuguese
Source: PLoS One. 2019 Apr 25;14(4):e0212303. doi: 10.1371/journal.pone.0212303 (PMC6483184; doi:10.1371/journal.pone.0212303)
Supplement: S1 Text — (PDF) [file pone.0212303.s001.pdf]

S1 Mathematical Details. In the model described in the text, for a fixed population of size N, we have some proportion ρN every time step who are L2 speakers. Each of these can 'mutate' (i.e. assign probability 1 to a variant *other* than the existing variant) with probability μ. Thus, every time step there will be $\mu\rho N$ mutants in the population.

In each round of interaction every individual will be assigned to an exclusive pair of interacting agents. Let us call the set of all such possible assignments for the population S. For a given $s \in S$, we will have three possible types of pairings (i, j):

1. Two non-mutants, whose probabilities of using the existing variant are p_{i1} and p_{j1}.
2. A non-mutant and a mutant, whose probabilities of using the existing variant are p_{i1} and 0, respectively.
3. Two mutants, whose probabilities of using the existing variant are both 0.

The change in the population mean frequency of the existing variant is equal to $\Delta\bar{p_1} = \frac{1}{N}\sum_{i=1}^{N}\Delta p_{i1}$, where $\Delta p_{i1} = p'_{i1} - p_{i1}$. Thus,

$$E[\bar{p_1}] = \frac{1}{N}\sum_{i=1}^{N} E[\Delta p_{i1}] = \frac{2}{N}\sum_{(i,j)\in s} E[\Delta p_{i1} + \Delta p_{j1}], \tag{1}$$

since every individual is assigned to an exclusive pair. For a pairing of type 1, we have.

$$E[\Delta p_{i1} + \Delta p_{j1}] = E[\Delta p_{i1}] + E[\Delta p_{j1}] \tag{2}$$

$$= (1 - p_{i1})lp_{j1} - p_{i1}l(1 - p_{j1}) + (1 - p_{j1})lp_{i1} - p_{j1}l(1 - p_{i1}) = 0 \tag{3}$$

For a pairing of type 2 (assuming i is the non-mutant), we have,

$$E[\Delta p_{i1} + \Delta p_{j1}] = E[\Delta p_{i1}] + E[\Delta p_{j1}] \tag{4}$$

$$= -lp_{i1} + lp_{i1} = 0 \tag{5}$$

And finally, for a pairing of type 3,

$$E[\Delta p_{i1} + \Delta p_{j1}] = 0 \tag{6}$$

$$\tag{7}$$

No matter which $s \in S$ we choose, the sum over all pairings is 0; thus $E[\Delta\bar{p_1}] = 0$, and the process is neutral with respect to interaction and the existing variant.
