# Supplementary material for: A model of contact-induced language change: Testing the role of second language speakers in the evolution of Mozambican Portuguese
Source: PLoS One. 2019 Apr 25;14(4):e0212303. doi: 10.1371/journal.pone.0212303 (PMC6483184; doi:10.1371/journal.pone.0212303)
Supplement: S2 Text — (PDF) [file pone.0212303.s002.pdf]

Maputo demographic data.

Table 1. Demographic data on Portuguese speakers in Maputo, Mozambique.

year	L1 speakers	L2 speakers
1975	100*	20,000
1980	6,525	326,521
1997	241,709	599,438
2007	470,690	612,992

We obtained demographic data on the number of L1 and L2 speakers of Portuguese in Maputo, Mozambique in the years 1975,1980,1997, and 2007 (*see* Table 1). This period is important because it captures the dramatic expansion of Portuguese speakers in Maputo following the official independence of the Republic of Mozambique in 1975, during which Mozambiquan Portuguese has undergone a number of changes. We assumed geometric growth between each time point and estimated the rates at which L1 and L2 speakers entered the population (b and r) in the following way:

1. Fix the overall growth rate (g) of the Portuguese speaking population based on the values in Table 1.

2. Calculate the recruitment rate, $r = g + d - b$.

The above method gave us demographic projections that matched the data very closely, and matched the proportions of L1 and L2 speakers at each time point particularly closely. We used these parameter estimates to simulate language change in Maputo according to the model describe above.

Table A. Estimated rate of recruitment of L2 speakers for Maputo Portuguese-speaking population.

period	recruitment rate
1975-1980	0.7590218
1980-1997	0.04677117
1997-2007	0.0128031
