# Supplementary material for: A model of contact-induced language change: Testing the role of second language speakers in the evolution of Mozambican Portuguese
Source: PLoS One. 2019 Apr 25;14(4):e0212303. doi: 10.1371/journal.pone.0212303 (PMC6483184; doi:10.1371/journal.pone.0212303)
Supplement: S3 Text — (PDF) [file pone.0212303.s003.pdf]

Linguistic data.
We use empirical data concerning two variable grammatical features: third person plural verbal agreement and choice of destination prepositions:

1. **Third person plural verbal agreement**: We include verbs where the subject is a plural pronoun or a plural noun phrase. The conservative variant (assumed to be used by all speakers at the time of independence and at the beginning of the simulation) consists in the verb exposing agreement with the plural subject by a plural suffix. The novel variant is the lack of the plural suffix on the verb.

2. **Destination prepositions**: We include contexts where the prepositions *a* (commonly *to* in English) or *em* (commonly *in* or *at* in English) are used to indicate destination, following a verb of movement or localization. The conservative variant consists in the use of the preposition *a*. The novel variant consists in the use of the preposition *em*. Before the introduction of this innovation, the preposition *em* has in locative contexts been restricted to indicating static spatial position.

The datasets come from recordings that were made in Maputo in 1993 and 2007 within the frameworks of studies by Stroud and Gonçalves (1997) and Jon-And (2011) respectively. Both samples consist in 20 recorded informal semi-structured sociolinguistic interviews of 30-60 minutes with 20 participants. The participants in the two samples are comparable in terms of age groups and education levels, factors that have often been found to determine linguistic variation. The recordings are made in similar circumstances and the interviews concern the same subject matters. All recordings were transcribed in order to identify conservative and novel linguistic variants.

For estimating the population mean probability of usage of innovative variants from the linguistic data, we assumed an individual's usage was binomially distributed. We further assumed that the parameter of that distribution was drawn from a beta distribution whose mean was the mean probability of usage in the population. We then used maximum likelihood to estimate the mean and confidence intervals of the population mean probability of usage for both innovative verb and preposition forms.

Table B. Linguistic usage data from Maputo Portuguese speakers.

Year	Third person plural verbal agreement		Destination prepositions	
	With plural marking	Without plural marking	*a*	*em*
1993/4	703	83	108	22
2007	638	160	159	55
